# COVID-19 pandemic response varies by clinical trial sponsor type

**DOI:** 10.1017/cts.2021.25

**Published:** 2021-03-16

**Authors:** Lisa Cooper, Irene Lee, Doreen Waldron Lechner

**Affiliations:** 1 Department of Health Informatics, School of Health Professions, Rutgers, The State University of New Jersey, Newark, NJ, USA; 2 Ernest Mario School of Pharmacy, Rutgers, The State University of New Jersey, Piscataway, NJ, USA

**Keywords:** COVID-19, SARS-Cov-2, Coronavirus, ClinicalTrials.gov, pandemic

## Abstract

**Introduction:**

The COVID-19 pandemic has impacted millions of lives globally. To learn more about this disease and find potential diagnostic, therapeutic, and preventative products, the healthcare community has initiated a staggering number of clinical trials.

**Methods:**

ClinicalTrials.gov was reviewed to determine if trial sponsor type had a relationship to time to COVID-19 response, which was defined as the date from disease discovery in Wuhan, China to ClinicalTrials.gov study “First Posted” date.

**Results:**

A total of 673 United States (US) sponsored, interventional study listings were retrieved, of which 293 (43.5%) were Industry-sponsored, 349 (51.9%) were Academic sponsored, and 31 (4.6%) were Other sponsor types. Of the Academic studies, 181 (51.9%) were Clinical and Translational Science Award (CTSA) hubs. The average response time for all sponsor types was 189 days, with Academic sponsors having the shortest average response time of 172.6 days (*P* < 0.001). CTSA hubs had a significantly (*P* < 0.001) shorter average response time (168.1 days) compared to all other sponsor types (197.4 days). However, while shorter in duration by 9.4 days, response time was not significantly different from non-CTSA sponsors (177.5 days; *P* = 0.238). Additionally, ANOVA indicated significant relationships (*P* < 0.001) between funding type, study phase, number of sites, and enrollment size on response time.

**Conclusions:**

Studies posted with the shortest response time were Academic-sponsored trials and included smaller sized investigations of repurposed approved or investigational drugs for the treatment of COVID-19 symptoms. A small second wave of study postings occurred approximately 4 months later, and included small, unique therapies targeting prevention or treatment of COVID-19.

## Introduction

The conclusion of 2020 marked nearly 82 million globally confirmed cases of COVID-19, the respiratory disease caused by the novel coronavirus, SARS-CoV-2 (or 2019-nCov) [1]. The disease, which was initially identified in Wuhan, China in December 2019, was declared a global pandemic by the World Health Organization (WHO) on March 22, 2020 and quickly spread throughout the world [2]. The virus is spread from person to person via respiratory droplets, with symptoms of disease ranging from asymptomatic to critical [3,4]. It is believed that underlying genetic predisposition and comorbidities, including advanced age, play a significant role in disease severity [5,6]. Initial treatment of COVID-19-infected patients focused on reducing symptoms and providing supportive care [4]. As the number of cases exponentially grew, the anticipated strain on healthcare resources, such as ICU beds, personal protective equipment (PPE), ventilators, and even healthcare personnel, resulted in the establishment of strict business, travel, and community guidelines by the Center of Disease Control (CDC), designed to slow the spread of disease [7,8].

To facilitate the expedited development of preventative, therapeutic, and diagnostic products to mitigate the global health crisis created by the COVID-19 pandemic, the Food and Drug Administration (FDA) shifted resource priorities and encouraged the use of established statutory programs. These recognized regulatory pathways included Emergency Use Authorization (EUA) [9], Expanded Access, Accelerated and Priority Approvals, and the more recently founded Breakthrough Designation program [10]. In addition to these programs, the repurposing of previously approved therapeutics, such as hydroxychloroquine which is approved for the treatment of malaria, and the utilization of already established nonclinical and clinical data for investigational products already in the clinic, can reduce the amount of time needed to initiate clinical investigations [11,12]. As a result, numerous diagnostic tests, PPE, and devices, such as ventilators, were quickly cleared or approved by the FDA [10]. Additionally, the first two vaccines (Pfizer, Inc./BioNTech, Collegeville, PA, USA and Moderna, Inc., Cambridge, MA, USA) for the prevention of COVID-19 infection were manufactured, investigated in the clinical setting, and approved under EUA in less than a year, a precedent-setting timeline [13,14]. The Moderna vaccine, along with other vaccines currently in development, was part of the Accelerating COVID-19 Therapeutic Interventions and Vaccines (ACTIV) initiative which began in April 2020, joining multiple agencies within the Department of Health and Human Services (HHS) together to streamline and speed up COVID-19 vaccine and therapeutic development [15].

ClinicalTrials.gov, is an online public registry of global clinical trials by the National Library of Medicine (NLM), a division under the National Institute of Health (NIH). The registry, which was publicly released in 2000, was created to increase access to clinical trials for a multitude of indications and their resulting data by patients, healthcare professionals, and researchers. Posting requirements are outlined in the Food and Drug Administration Modernization Act of 1997 (FDAMA), and excludes the need to post certain studies, such as observational and Phase 1 healthy volunteer studies. Trial sponsors conducting studies under an investigational new drug (IND) application must submit certification of compliance with these requirements (Form FDA 3674) and postings must be made prior to enrolling the first subject. This certification requirement went into effect in 2007 under 2 U.S.C. § 282(j)(5)(B), section 402(j)(5)(B) of the Public Health Service Act. As a condition of publication, the International Committee of Medical Journal Editors (ICMJE) began requiring the posting of clinical trials in the registry in 2005, which has prompted sponsors to post studies regardless of regulated posting requirements. As of the close of 2020, more than 360,000 trials have been posted to the registry [16,17].

The Code of Federal Regulations, Title 21 (21 CFR), requires study sponsors to conduct clinical investigations within the United States (US) under an IND application. These applications are reviewed by the FDA and a determination of approval is provided to the application sponsor within 30 days [18]. In some cases, clinical studies may be conducted under an already activated IND for the same investigational product; however, FDA has indicated that, in the case of COVID-19 trials, this is not preferential. Additionally, the FDA has encouraged COVID-19 trial sponsors to seek pre-IND guidance to help ensure a more efficient and expeditious IND review, and released a Guidance for Industry and Investigators in May 2020 supporting this initiative [19,20]. A sponsor is defined in 21 CFR 312 as an “individual, pharmaceutical company, government agency, academic institution, private organization, or other organization” [18]. At the time of submission of an IND, the sponsor must indicate if the IND is for “commercial” or “research” purposes. By selecting commercial, the sponsor is indicating that the product being developed is intended to be commercialized at a later date [21].

In addition to approval by the FDA, each study site must obtain approval from its governing Institutional Review Board (IRB). In general, these IRBs must comply with the requirements set out in 21 CFR 56, which includes a review of study documents, such as the protocol and informed consent forms, to ensure that the level of risk to the patient has been minimized and that the study procedures and design are sound [22]. Most institutions (95%) have their own IRB; however, in some cases, a central IRB may be used. The timing for review by the IRB is unique to each institution. The Association for the Accreditation of Human Research Protection Programs (AAHRPP) found that full approval by US IRBs took an average of 37 calendar days [23]; however, longer review durations are not unexpected [24].

Academic institutions play a significant role in furthering the advancement of therapies. In addition to participation in Industry-sponsored studies as clinical research sites, Academic institutions often play the role of sponsor, designing and conducting their own research. The Association for Clinical and Translational Sciences (ACTS) was created in 2009 to facilitate timely translational and clinical research. Additionally, the National Center for Advancing Translational Sciences (NCATS), a division under the NIH, has a similar mission of improving health through advancing translational science. One way both groups work to achieve their mission is through supporting the Clinical and Translational Science Awards (CTSA) program. This program provides funding for the development and execution of innovative, collaborative, and streamlined research processes and conduct. During the year 2020, the CTSA program consisted of over 57 CTSA hubs, institutions receiving CTSA support, across the US [25].

Considering the types of studies Academic and Industry sponsors were likely to conduct, as well as the likelihood of program delays caused by the need for manufacturing, generation of nonclinical data, and pre-IND meetings, we hypothesized that Academic sponsors would sponsor more research-focused studies and have a quicker response time to the COVID-19 pandemic compared to the pharmaceutical industry (Industry) and Other sponsor types.

## Materials and Methods

ClinicalTrials.gov was searched on January 1, 2021 using the terms “COVID-19” and “SARS-Cov-2.” The scope of the search results was further restricted to include only “Interventional” studies, those which had a “Condition” of at least one of the following terms: “COVID,” “COVID-19,” “Coronavirus,” “SARS-Cov-2,” “SARS,” or “2019-nCoV,” and whose primary sponsor is located in the US or had at least one US trial site. Behavioral studies were removed, as were listings posted prior to December 31, 2019 (last day of the month COVID-19 was discovered in Wuhan, China). Study sponsors were then categorized based on “Sponsor” information within each study listing as (1) Industry, (2) Academic, or (3) Other (e.g., government and nonprofit organizations such as the NIH and Bill and Melinda Gates Foundation, respectively). Academic sponsors were further identified as CTSA hubs (or affiliates) or non-CTSA hubs. Response time for each listing was determined by calculating the number of days from December 31, 2019 to the “First Posted” date (date the listing was first posted to ClinicalTrials.gov by the study sponsor).

Descriptive statistics were used to analyze listing demographics. ANOVA was used to determine the significance of sponsor type, funding type, study phase, number of sites, and enrollment size as independent variables on response time.

## Results

**Fig. 1.**
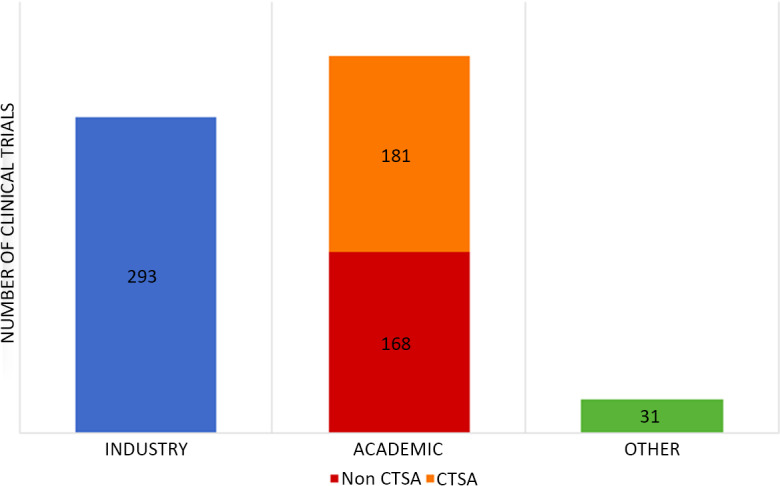
Clinical trial by sponsor type.

A total of 673 listings were utilized as the final analysis dataset. Of these, 293 (43.5%) were Industry-sponsored, 349 (51.9%) were sponsored by an Academic institutions, and 31 (4.6%) were Other sponsor types (see Fig. 1). Of the Academic-sponsored studies, 181 (51.9%) were CTSA hubs.

Overall, 91 (13.5%) Phase 1, 53 (7.9%) Phase 1/2, 259 (38.5%) Phase 2, 38 (5.6%) Phase 2/3, 98 (14.6%) Phase 3, 31 (4.6%) Phase 4, and 103 (15.3%) undefined phase studies were listed. More studies, 307 (45.6%), expected to enroll ≤99 subjects, compared to 233 (34.6%) studies which were to enroll 100–500 subjects, and 126 (18.7%) anticipated to enroll ≥501 subjects. Seven (1.1%) study listings did not specify expected enrollment. A total of 540 (80.2%) listings included study site information, and of these, 308 (57.0%) included only a single site, 111 (20.5%) had 2–5 sites, 38 (7.0%) had 6–10 sites, 56 (10.4%) had 11–50 sites, 17 (3.1%) had 51–100 sites, and 10 (1.9%) had ≥101 sites. Study phase, enrollment, and the number of sites by sponsor type are presented in Table 1. Industry funding supported 271 (40.3%) trials, with government funding only 20 (2.9%) listed trials. The remaining 382 (56.8%) of trials had primary funding listed as Other.

**Table 1.** Study phase, enrollment size, and number of sites by sponsor type

	Industry (*n* = 293)	Academic (*n* = 349)	Other (*n* = 31)
Study phase			
Phase 1	47 (16.0%)	41 (11.7%)	3 (9.7%)
Phase 1/2	30 (10.2%)	19 (5.4%)	4 (12.9%)
Phase 2	109 (37.2%)	142 (40.6%)	8 (25.8%)
Phase 2/3	24 (8.2%)	12 (3.4%)	2 (6.5%)
Phase 3	43 (14.7%)	45 (12.9%)	10 (32.3%)
Phase 4	6 (2.0%)	24 (6.9%)	1 (3.2%)
Undefined	34 (11.6%)	66 (18.9%)	3 (9.7%)
Enrollment			
<99	131 (44.7%)	169 (48.4%)	7 (22.6%)
100–500	101 (34.5%)	124 (35.5%)	8 (25.8%)
≥501	54 (18.4%)	56 (16.0%)	16 (51.6%)
Not specified	7 (2.4%)	0 (0.0%)	0 (0.0%)
Sites			
1	72 (24.5%)	226 (64.8%)	10 (32.2%)
2–5	52 (17.7%)	55 (15.8%)	4 (12.9%)
6–10	24 (8.2%)	11 (3.2%)	3 (9.7%)
11–50	41 (14%)	10 (2.9%)	5 (16.1%)
51–100	12 (4.1%)	1 (0.3%)	4 (12.9%)
≥101	10 (3.4%)	0 (0%)	0 (0%)
Not specified	82 (28%)	46 (13.2%)	5 (16.1%)

The average response time for all sponsor types (*n* = 673) was 189 days. Academic sponsors had the shortest average response time, 172.6 days, which was statistically significant compared to Industry (208.5 days) and Other sponsor types (199.5 days) (*P* < 0.001). CTSA hubs had a significantly (*P* < 0.001) shorter average response time (168.1 days) compared to all sponsor types excluding CTSA hubs (197.4 days), as well as compared to Industry sponsors (*P* < 0.001); however, while shorter in duration by 9.4 days, CTSA hub average response time was not significant compared to non-CTSA Academic sponsors (177.5 days; *n* = 168; *P* = 0.238). Non-CTSA hub Academic sponsors had a significantly shorter average response time compared to Industry sponsors (*P* < 0.001). Due to the limited number of Other sponsor types, additional analysis was not conducted on response time.

**Fig. 2.**
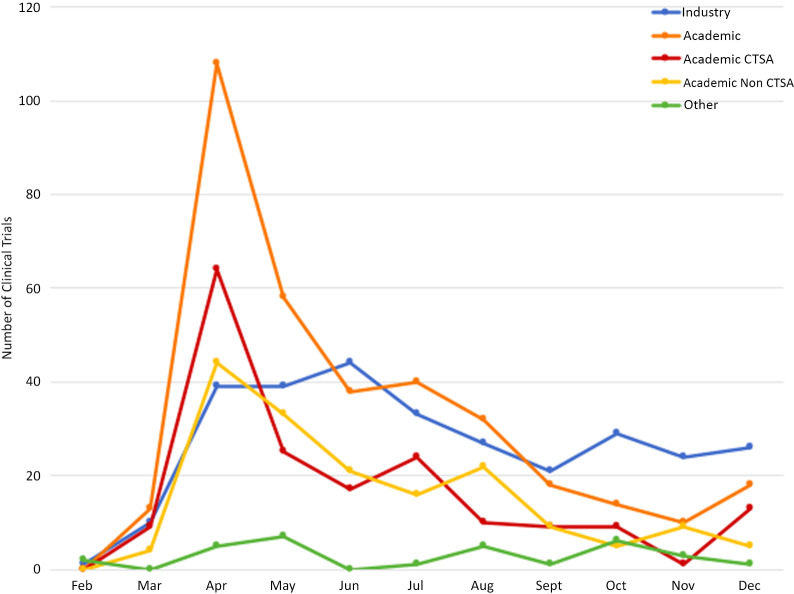
Number of clinical trials by first posted date versus sponsor type.

For Academic sponsors, the largest number of study postings to ClinicalTrials.gov occurred in the month of April 2020 (*n* = 109), of which 63 (57.8%) listings were by CTSA hubs and 46 (42.2%) listings were by non-CTSA hubs. Following April, the number of Academic postings sharply declined each month, with only a slight uptick in July (*n* = 40). Industry sponsors had the largest number of study posts in June 2020 (*n* = 44), with a more gradual decline each month, and a slight uptick in October (*n* = 29; Fig. 2).

Study phase significantly impacted average response time (*P* < 0.001) with Phase 2 studies having the shortest response time (172.6 days), followed by Phase 3 studies (177.5 days), Phase 2/3 studies (195.1 days), Phase 1/2 studies (200.1 days), Phase 4 studies (203.2 days), and lastly, Phase 1 studies (211.5 days). Enrollment size also significantly impacted average response time (*P* = 0.005). Studies enrolling 100–500 subjects had the shortest average response time of 174.6 days, followed by studies enrolling <99 subjects (193.9 days) and studies enrolling >501 subjects (199.1 days). The number of study sites also had a significant impact on average response time, with study listing which contained 11–50 sites having the shortest average response time of 146.7 days. Studies with 51–100 sites had the second shortest average response time of 154 days, followed by studies with 6–10 sites (169.1 days), >101 sites (174.8 days), 2–5 sites (181.2 days), 1 site (183.9 days), and those listings with no sites identified (229.7 days). Finally, average response time was significantly impacted by funding type (*P* < 0.001), with an average response time of 222.8 days for Industry-funded studies, 193 days for government-funded studies, and 190.9 days for studies with funded listed as Other.

## Discussion

The majority of COVID-19 clinical studies listed within ClinicalTrials.gov were smaller, earlier phase studies (Phases 1–2), with either a single site or limited number of sites (< 5), and were sponsored predominately by Academic and Industry sponsors. While both Academic and Industry sponsors initiated studies repurposing drugs previously approved (marketed) by the FDA for other indications, this strategy accounted for the larger portion of early study listings by Academic sponsors, a trend seen for CTSA hub and non-CTSA hub sponsors. Industry sponsors had a proportional mix of repurposed previous approved therapies and repurposed novel therapy studies which were already being investigated for other indications. In some cases, clinical studies investigating already approved therapies may be exempt from the requirement of being conducted under an IND [18]; eliminating the time constraints posed by IND submission, review, and approval processes. While repurposed novel therapies would require an IND, sponsors may determine there is no need for a pre-IND meeting with the FDA since manufacturing, nonclinical, and early clinical data would already have been established. Additionally, it is important to note that many of these early studies posted by both Academic and Industry sponsors were designed as interventional studies for the treatment of COVID-19 symptoms, such as respiratory distress, rather than prevention or treatment of the disease itself. In contrast, Other sponsor types had more larger sized, later stage (Phase 3 and Phase 4) studies as a percentage of their overall posted studies. These Other-sponsored studies predominantly involved investigations of hydroxychloroquine and remdesivir, and mostly were funded by the NIH. This combination of study complexity due to study size and navigating bureaucratic requirements for funding likely play a role in the longer response duration for Other sponsor types.

After the initial influx of studies posted to ClinicalTrials.gov, in April for Academic sponsors and in June for Industry sponsors, a second minor bolus of studies was posted approximately 4 months later, in August for Academic sponsors and in October for Industry sponsors. In these later studies, Academic sponsors continued to focus on repurposed marketed drugs; however, Industry sponsors posted more repurposed novel therapy investigations compared to repurposed marketed drugs. These investigations included several early phase (Phases 1 and 2) studies focused on the treatment of COVID-19, rather than only symptom management. This shift by Industry sponsors is likely a result of the completion of data to support the opening of an IND [18]. The time it takes to generate this required information varies, and is influenced by company experience, type of nonclinical tests needing to be performed, type of product being developed, and the clinical indication the product is intended to be used for [26,27]. Pre-IND meetings with the FDA are more likely for these types of studies/development programs, and are encouraged by the FDA; however, the time it takes to request and hold such a meeting, even with the FDA’s consolidated process for COVID-19 programs, would subsequently increase the time to IND submission to FDA; thereby increasing the time from developing the study concept to posting on ClinicalTrials.gov [19,20,28].

Study postings to ClinialTrials.gov typically occur subsequent to the opening of the IND, as the IND provides greater assurance that the study will be allowed to be conducted and the listing details will not need immediate revision due to protocol amendments resulting from IND application review comments. Posting date does not account for the time from IND approval to actual study start which could be quite variable. At the time of posting, sponsors estimate their study start date, which is determined by several factors, including site IRB approval, site training, and drug availability [29]. As this start date is only a sponsor’s best estimate at the time of initial study posting and may have not actually occurred yet and therefore could be inaccurate, the study posting date was used to determine the time to response.

Limitations of this pandemic response analysis are mostly accounted for by the quality of information with the study postings. While all postings reviewed by the ClinicalTrials.gov administrators prior to releasing the posting to the public [16], the information contained in the posting is vastly dependent on the sponsor’s willingness to disclose and accurately record information. Several listings within the dataset had incorrect or missing data elements. For example, several listings did not provide any, or only provided minimal site location information. This may be because sites were not yet identified by the sponsor at the time of initial posting, or it may be a strategy to avoid disclosing site locations to potential competitors. Additionally, some sponsors listed COVID-19 as a “condition” when the study, in fact, was not evaluating COVID-19. In these instances, the listings were removed from the dataset when identified. Despite these challenges, the number of studies evaluated and information contained within the listings were determined to be robust enough to ensure true findings.

The results of the analysis support the hypothesis that Academic sponsors had a quicker response time to the COVID-19 pandemic compared to all other sponsor types. Given the types of trials Academic sponsors were conducting, it is not surprising they had a nearly 36-day faster response time compared to Industry sponsors. Type of IND (commercial vs. research) should be assessed for impact on response time in future analysis. Additionally, controlling for sponsor collaborators should also be considered as some Academic sponsors are conducting Industry-driven studies.
